# Anterior Rectal Resection in a Patient with Aortoiliac Occlusive Disease and Coexisting Collateral Pathways: Management and Pitfalls

**DOI:** 10.1155/2016/3721260

**Published:** 2016-10-09

**Authors:** Floryn Cherbanyk, Jean-Loup Gassend, Olivier Martinet, Snezana Andrejevic-Blant, Henri-Marcel Hoogewoud

**Affiliations:** ^1^Department of Surgery, Riviera-Chablais Hospital, 1820 Montreux, Switzerland; ^2^Laboratory of Pathology and Cytology Unilabs, CYPA-Lausanne, 1066 Epalinges, Switzerland; ^3^Department of Radiology, HFR Fribourg, Fribourg, Switzerland

## Abstract

Chronic aortoiliac occlusive disease most often affects the common iliac arteries and distal aorta but can progress all the way to the renal arteries, occluding the inferior mesenteric artery. A compensatory collateral network typically develops to preserve lower body perfusion. Inadvertent compression or ligation of such collaterals during surgery can have catastrophic consequences. In this article, we present the case of a 63-year-old patient with aortoiliac occlusive disease, requiring surgery for an adenocarcinoma of the rectosigmoid junction. A CT angiography was performed in order to map out the collateral pathways that had developed and Doppler ultrasound was used to mark their positions. The surgical procedure was adapted to his specific anatomy. A successful anterior resection was performed, and the patient made an uneventful recovery. In cases of aortoiliac obliteration, the existence of collaterals must be kept in mind and investigated with a multidisciplinary approach before any surgery is considered.

## 1. Introduction

Colorectal cancer and chronic infrarenal aortic thrombosis may coexist, particularly in elderly patients. Abdominal aortic occlusion is most commonly a chronic condition caused by atherosclerotic plaque at the aortic bifurcation. Chronic thrombotic obliteration most often affects the common iliac arteries and distal aorta but can progress all the way to the renal arteries, occluding the inferior mesenteric artery. The prevalence of the disease is not known because rich collateral networks develop, ensuring blood flow to the lower body and enabling many patients to remain asymptomatic [1, 2]. One common collateral, known as Winslow's pathway, carries blood through the mammary arteries to the epigastric arteries and then into the external iliac arteries. As the vascular anatomy may be greatly modified in cases of aortic thrombosis, the ligation of certain blood vessels which is considered routine surgical procedure may have catastrophic effects. To avoid such negative outcomes, a CT angiography should be performed preoperatively, so that the patient's specific anatomy can be precisely mapped out, in order for the surgical procedure to be adapted in consequence [3].

## 2. Case Presentation

In the following report, we discuss the case of a 63-year-old patient who presented with a five-month history of constipation that was not relieved by laxatives. The patient, a smoker, was known to have type II diabetes, chronic hypertension, and hypercholesterolemia. He was equipped with prosthetic limbs after having suffered a traumatic, below the knee, amputation of both lower limbs during his youth.

On clinical examination at admission, blood pressure was 135/85 mmHg, pulse was 80/min, blood oxygen saturation was 98%, and temperature was 36,8°C. The femoral pulses were palpable. Abdominal auscultation was unremarkable, but the left iliac fossa was painful upon palpation. There was no history of nausea or vomiting.

Blood tests were normal, including the tumor markers carcinoembryonic antigen (CEA) and carbohydrate antigen 19-9 (CA-19-9).

The patient then underwent a colonoscopy that revealed the presence of a stenosing mass of the rectosigmoid junction, 18 cm from the anal margin, which was biopsied and determined to be an adenocarcinoma.

**Figure 1:**
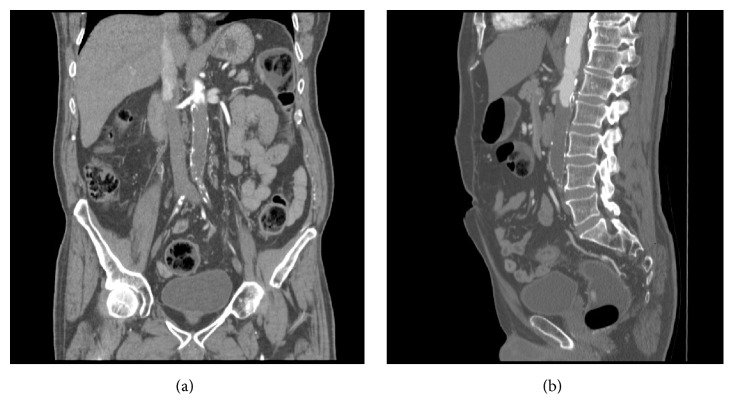
Coronal and sagittal CT angiography views, showing complete thrombosis of the aortoiliac axis.

A thoracoabdominal CT scan performed for assessment of disease extension revealed that the patient suffered from chronic aortoiliac occlusive disease. A CT angiography was therefore performed, which showed that the distal aorta was completely occluded, with the occlusion starting immediately distal to the renal arteries and also involving the inferior mesenteric artery (Figures 1(a) and 1(b)).

**Figure 2:**
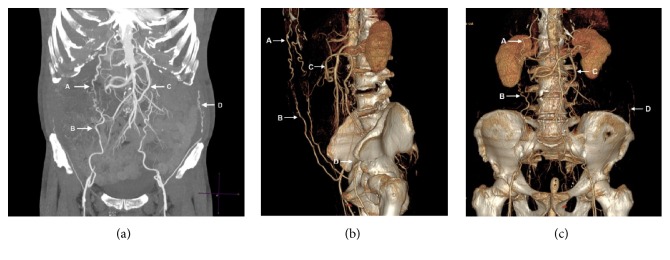
CT angiography and 3D reconstruction showing Winslow's pathway (A and B), the arc of Riolan (C), and the lower intercostal artery to circumflex iliac artery collateral route (D).

Circulation to the legs occurred through collaterals involving the mammary and epigastric arteries (Winslow's pathway) [4, 5], as well as the lower intercostal and subcostal arteries and the external iliac artery via the circumflex iliac artery. Blood was supplied to the colon through the arc of Riolan and the marginal artery (of Drummond). By retrograde flow, these also provided blood to the inferior mesenteric artery up to 2 cm distally of its connection with the thrombosed aorta (Figure 2).

**Figure 3:**
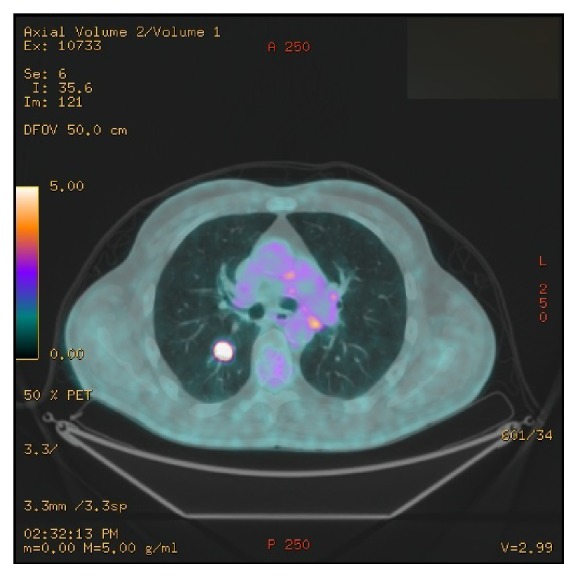
18F-FDG PET-CT scan showing a highly suspicious lesion in the right lung.

Furthermore, the CT scan showed the presence of a probable metastasis in the inferior lobe of the right lung. An 18F-FDG PET-CT scan confirmed the highly suspicious nature of the lesion in the right lung (Figure 3).

**Figure 4:**
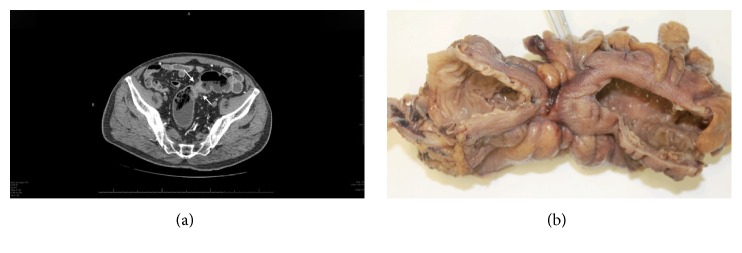
Axial CT scan view and postoperative specimen illustrating the occlusive nature of the tumor.

**Figure 5:**
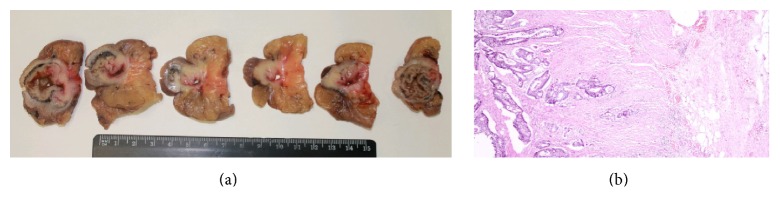
Macroscopic appearance and histology views of the tumor, showing occlusive moderately differentiated colonic adenocarcinoma.

Considering the occlusive nature (Figures 4(a) and 4(b)) of the adenocarcinoma, the patient needed rapid surgical intervention. The CT imagery was reviewed by the radiologist and the surgeon together. The day before the operation, the radiologist marked the positions of the epigastric arteries using Doppler ultrasound, after which the stomatotherapist determined the best site for the protective ileostomy. The surgeon then performed the planned rectosigmoid resection. A colorectal terminoterminal anastomosis was created with a circular stapler and protected with a right protective ileostomy. The postoperative period was complicated by a minor surgical site infection. Pathological TNM staging classified the tumor as pT3, pN1a (1/10), G2-3, Pn1, and R0 (Figures 5(a) and 5(b)).

A percutaneous biopsy of the right lung lesion performed under CT guidance showed the presence of adenocarcinomatous cells compatible with a colic origin. Two months after surgery, the patient underwent 12 cycles of systemic chemotherapy with FOLFOX (folinic acid, 5-fluorouracil, and oxaliplatin), followed by five stereotaxic radiotherapy cycles for the pulmonary metastasis with a total of 55 Gy, with good effect.

The ileostomy was closed one year after the initial surgery. The patient's vascular status was unchanged, and he made an uneventful recovery.

Follow-up investigations (CT examination and colonoscopy) performed 6 and 12 months after the oncological treatment did not show any recurrence. Lab tests showed no increase of tumor markers.

## 3. Discussion

Chronic atherosclerotic disease obstructing the aorta and iliac arteries is often encountered in elderly patients. In such cases, the lower limbs and the distal digestive tract are typically perfused by networks of collaterals, such as Winslow's pathway. Though the incidence is not known, such vascular abnormalities can coexist with lower bowel cancer requiring surgery [6]. In such cases, collaboration between the radiologist and surgeon is of prime importance. If the surgeon is not aware of these patients' modified anatomy, the risk of damaging the collaterals and compromising blood supply to the lower extremity increases greatly [5, 7, 8].

In a series of 58 resections of the rectum, Manjoney et al. report that two patients developed lower limb ischemia requiring amputation, both of which had preexisting arterial disease [7]. Devine et al. and Perricone et al. have therefore advised that all patients requiring left colon or rectum resection should first be evaluated for aortoiliac occlusive disease [9, 10]. A high level of suspicion is needed, as even with complete aortic obstruction femoral pulses may still be palpable, as in the case reported in this paper. Currently, as CT scans are part of the standard diagnostic procedures for lower bowel cancer, major vascular abnormalities should be noticed by the radiologist before any surgery is performed.

**Figure 6:**
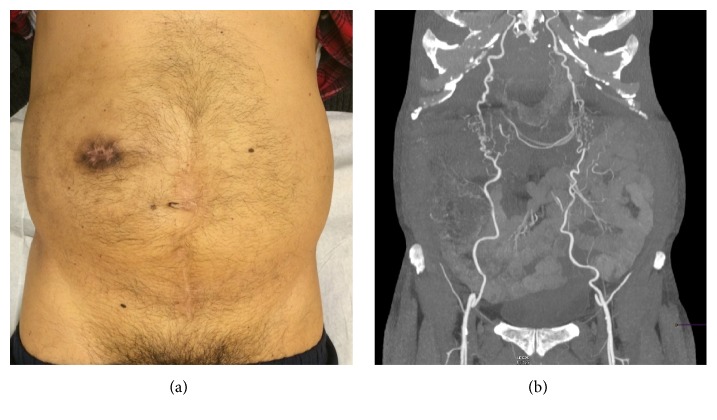
Postoperative view of the patient's abdomen, compared to a preoperative CT angiography, showing the proximity of Winslow's pathway and of the ileostomy.

The patient described here suffered from chronic aortoiliac occlusive disease, with the aorta distal to the renal arteries and the proximal femoral arteries being completely occluded. Lower limb perfusion occurred through Winslow's pathway (in which blood flows through the mammary arteries to the epigastric arteries and then into the external iliac arteries) and through the lower intercostal and subcostal arteries and the external iliac artery via the circumflex iliac artery. The arc of Riolan and the marginal artery provided blood to the lower GI tract and to the inferior mesenteric artery, by retrograde flow, up to 2 cm distally to the occluded aorta. In such a context, interruption of blood flow through the epigastric arteries or the marginal artery can have catastrophic consequences, including intestinal ischemia with anastomotic leakage, lower limb amputation, and death [10, 11]. Interruption of blood flow through the epigastric arteries not only can be caused by direct damage while installing the ileostomy (Figures 6(a) and 6(b)) but also can have less obvious causes, such as thrombosis following overzealous retraction or bad positioning of the patient. The surgical team should therefore be thoroughly briefed by the radiologist regarding the patient's specific anatomy, so that the surgical procedure can be adapted to the patient. This must be done without compromising the quality of the anastomosis or of the final oncological result. A multidisciplinary approach involving the radiologist, the surgeon, and the oncologist is therefore required.

## 4. Conclusion

Atherosclerotic disease is common in the elderly and can lead to complete chronic infrarenal aortic obstruction, with networks of collaterals, such as Winslow's pathway, supplying blood to the large bowel and lower limbs. These pathways may be clearly identified and evaluated with imaging methods, most notably CT angiography, and can be marked using Doppler ultrasound. The radiologist must be aware of all the known collateral pathways in order to correctly identify them. Collaboration between the radiologist and the surgical team is then essential in order to avoid the dangers of surgical manipulation of these collateral pathways.
